# Supplementary material for: Targeting Proliferating Tumor-Infiltrating Macrophages Facilitates Spatial Redistribution of CD8+ T Cells in Pancreatic Cancer
Source: Cancers (Basel). 2022 Mar 14;14(6):1474. doi: 10.3390/cancers14061474 (PMC8946118; doi:10.3390/cancers14061474)
Supplement: Supplementary file 1 [file cancers-14-01474-s001.zip › cancers-1588172-supplementary.pdf]

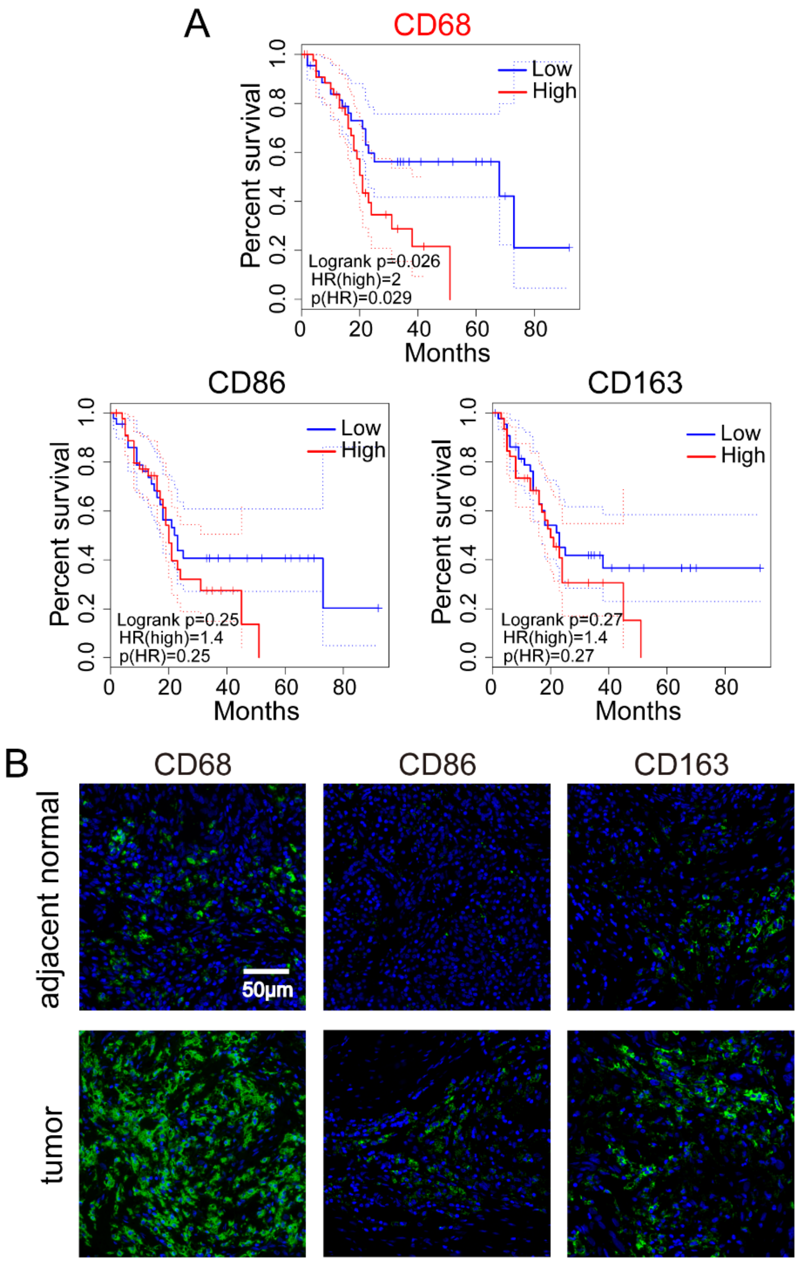

Figure S1. High infiltration of macrophages in PDAC is related to the clinical outcome. (**A**) Overall survival curve analysis based on CD68, CD86 and CD163 TPM expression in 179 pancreatic tumor cases from the GEPIA database. The “High” and “Low” group cutoff was set as the quartile (cutoff-high (%) was 75%, and cutoff-low (%) was 25%). n (high)=45, n (low)=45. (**B**) Representative IHC images of human PDAC and adjacent normal tissue assessed for CD68, CD86 and CD163 cells.

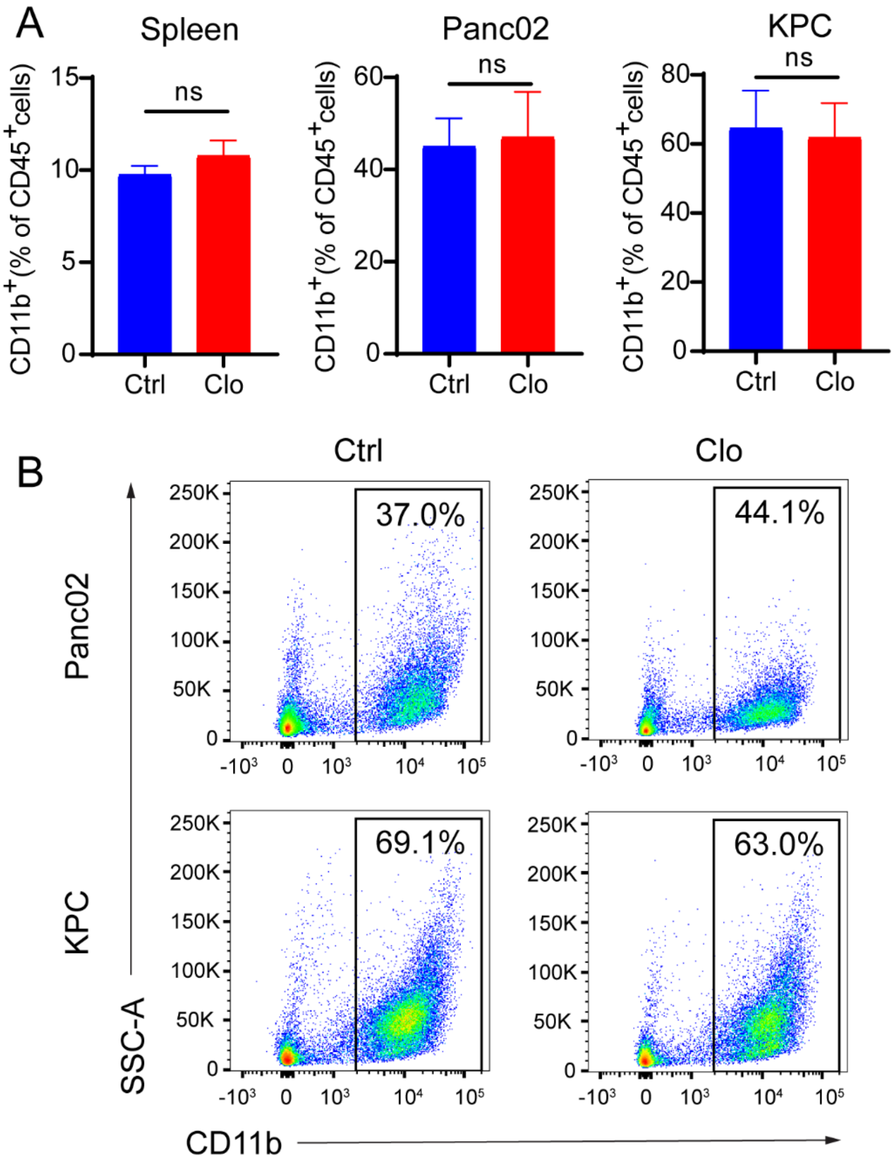

Figure S2. The abundance of CD11b⁺ cells did not show difference with or without clodronate liposomes treatment. (**A**) Statistical diagrams of CD11b⁺ myeloid cells on live CD45⁺ cells in spleens, Panc02 and KPC tumor tissues treated with or without clodronate liposomes. (**B**) Cells were isolated from murine tumor tissues and analyzed by flow cytometry. For all dot plots, live cells were selected by zombie negative cells, after gating on FSC-A and FSC-H which can excluded adhesive cells, CD45⁺ subpopulations were selected for further analyzed.

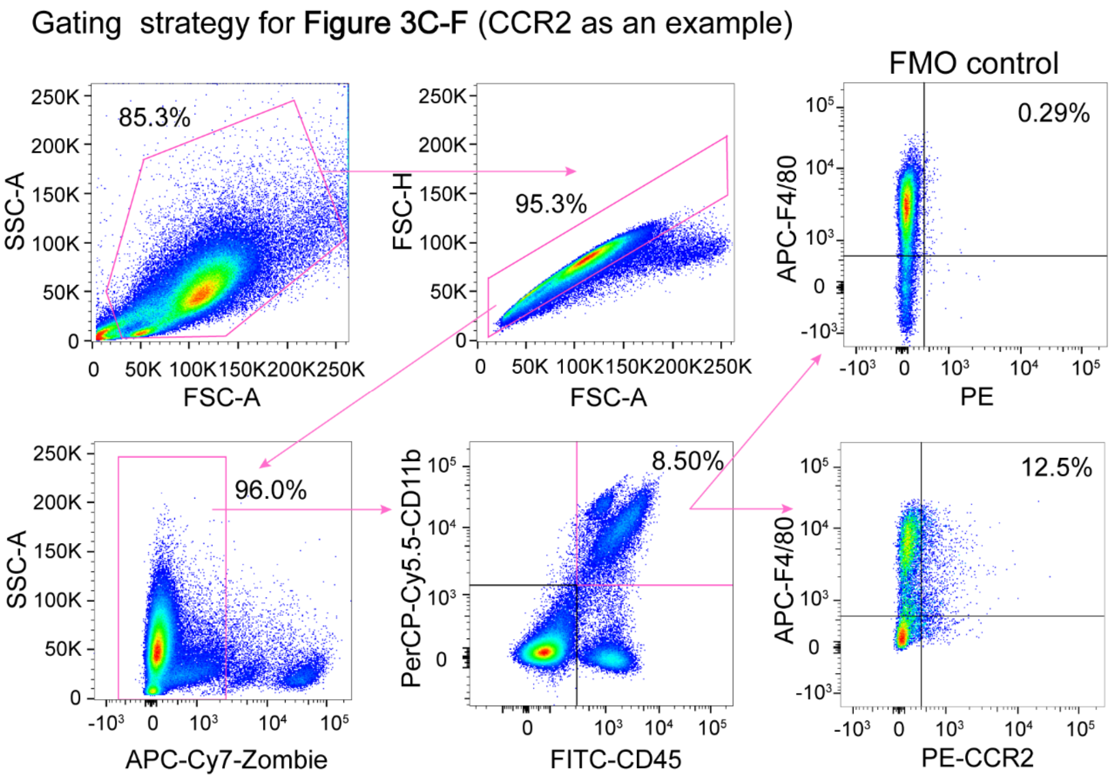

Figure S3. The gating strategy for macrophage subpopulations analysis (Figure 3C-F), and the FMO control (for PE channel) was used to determine the gate of macrophage subsets.

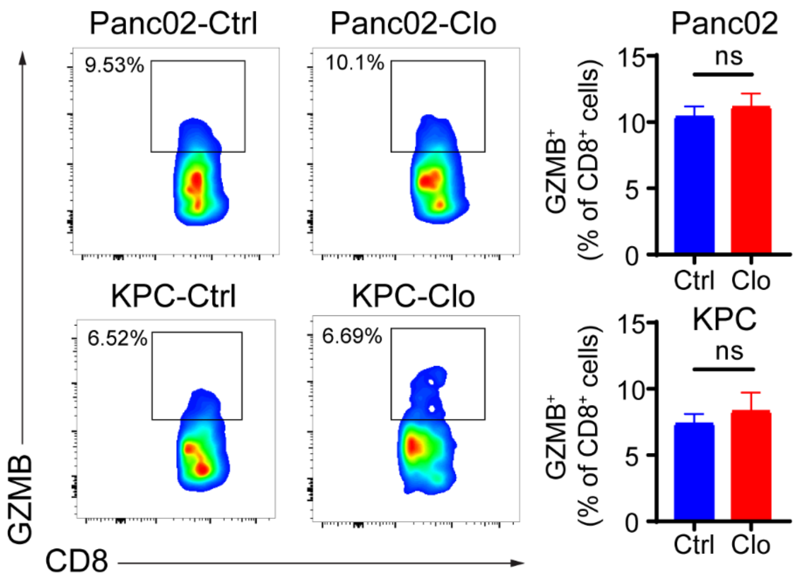

Figure S4. Flow cytometry analysis of granzyme B (GZMB) expressing CD8+ T cells in the TME of Panc02 and KPC tumors treated with PBS liposomes or clodronate liposomes.
